# Evaluation of medication safety assessment tools for pharmacist-led medication reviews: the Eastern European pilot project

**DOI:** 10.3389/fphar.2024.1348400

**Published:** 2024-02-16

**Authors:** Anita Tuula, Piotr Merks, Magdalena Waszyk-Nowaczyk, Mariola Drozd, Galina Petrova, Reka Viola, Veera Bobrova, Michael Scott, Marje Oona, Daisy Volmer

**Affiliations:** ^1^ Institute of Pharmacy, Faculty of Medicine, University of Tartu, Tartu, Estonia; ^2^ Department of Pharmacology and Clinical Pharmacology, Faculty of Medicine, Collegium Medicum, Cardinal Stefan Wyszyński University in Warsaw, Warsaw, Poland; ^3^ Pharmacy Practice Division, Chair and Department of Pharmaceutical Technology, Poznan, Poland; ^4^ Department of Humanities and Social Medicine, Medical University of Lublin, Lublin, Poland; ^5^ Faculty of Pharmacy, Medical University of Varna, Varna, Bulgaria; ^6^ University of Szeged, Faculty of Pharmacy, Department of Clinical Pharmacy, Szeged, Hungary; ^7^ Medicines Optimisation Innovation Centre, Antrim, Northern Ireland, United Kingdom; ^8^ Institute of Family Medicine and Public Health, Faculty of Medicine, University of Tartu, Tartu, Estonia

**Keywords:** medication use review, PIM list, adverse drug events, polypharmacy, community pharmacy services, Eastern Europe

## Abstract

**Background:** Pharmacist-led medication reviews (MR) are one of the key methods to support medication safety in polypharmacy patients. The aims of this study were to pilot MRs in Eastern European community pharmacies, describe medication use in polypharmacy patients, and evaluate the usability of medication safety assessment tools.

**Methods:** The MR pilot was undertaken in Estonia, Latvia, Poland, Hungary, Romania, and Bulgaria. Patients who used at least five medicines were directed to the service by their GPs. Data on drug-related problems (DRPs) and adherence were collected by pharmacists through structured patient interviews. Databases for identification of potential drug-drug interactions (pDDIs) and adverse drug reactions (ADRs) named Inxbase/Riskbase, as well as an integrated tool comprising potentially inappropriate medicines (PIMs) lists EU(7)-PIM and EURO-FORTA, were applied retroactively to the MR pilot data to investigate possibilities for their use and to describe medication use and potential risks in the study population.

**Results:** A total of 318 patients were included in the study, 250 of them elderly (≥65 years). One hundred and eighty (56.6%) participants had a total of 504 pDDIs based on Inxbase analysis. On average, there were 1.6 pDDIs per participant. Twenty-five (5.0%) of the 504 pDDIs were in a high-risk category. A total of 279 (87.7%) participants had a potential ADR in at least one of 10 Riskbase categories. One hundred and fifty-four (20.8%) of the potential ADRs were in a high-risk category. Twenty-seven pDDIs and 68 ADRs documented as DRPs during the service were not included in the databases. Using the integrated EU(7)-PIM/EURO-FORTA PIM list, a total of 816 PIMs were found in 240 (96%) of the 250 elderly participants (on average 3.4 PIMs per elderly participant). Seventy-one (29.6%) of the participants were using high-risk PIMs. Twenty-one percent of high-risk PIMs and 13.8% of medium-risk PIMs were documented as DRPs by the pharmacists during the pilot.

**Conclusion:** Medication safety assessment tools can be useful in guiding decision-making during MRs; however, these tools cannot replace patient interviews and monitoring. Tools that include a thorough explanation of the potential risks and are easy to use are more beneficial for MRs.

## 1 Introduction

The World Health Organization (WHO) considers medication reviews (MR) one of the key strategies to promote medication safety in polypharmacy patients (World Health Organization, 2019). Polypharmacy, most commonly defined as the concurrent use of five or more medicines (Masnoon et al., 2017), is a global health concern (World Health Organization, 2019). Polypharmacy patients are known to have lower responses to treatments and experience serious adverse events, leading to higher rates of mortality, morbidity, and hospitalization, mostly in older adults (Lu et al., 2015; Masnoon et al., 2017; Bechman et al., 2019). While concurrent treatment with multiple medicines is generally necessary in multimorbid patients, it is important to differentiate and reduce inappropriate polypharmacy where medicines are prescribed irrationally, e.g., when there is an unacceptably high risk of serious adverse reactions, sometimes with little health benefit, or when prescribed medicines fail to achieve treatment goals, often due to the patient’s inability or unwillingness to use them correctly (Scottish Government Model of Care Polypharmacy Working Group, 2015). In addition to adverse clinical outcomes, inappropriate prescribing and polypharmacy lead to higher healthcare costs, thereby demanding nationwide action (Cahir et al., 2010).

Inappropriate polypharmacy often leads to adverse drug events (ADEs). ADEs are considered a major burden on the healthcare system, with more than two-thirds of such events considered preventable with adequate patient instructions, monitoring, follow-up, and reassessment after changes in treatment regimen (Woo et al., 2020). Older patients especially are at higher risk of ADEs, as aging is associated with changes in both pharmacokinetics and pharmacodynamics (Hutchison and O’Brien, 2007). In elderly patients, the use of potentially inappropriate medicines (PIMs), i.e., medicines that should be avoided in older people because of the potential adverse effects outweighing the benefits, increases the risk of the onset of geriatric syndromes, especially falls, frailty, and functional and cognitive impairment (Muhlack et al., 2018; Kucukdagli et al., 2020).

The WHO guidelines recommend that health workers always consider cessation of medicines when conducting MRs (World Health Organization, 2019). Deprescribing is the planned and supervised process of stopping a medicine or tampering doses in the event that the medicine is causing more harm than good (Deprescribing, 2024). Community pharmacists as specialists in pharmacotherapy are valuable partners in deprescribing and could lead the process through interventions such as MRs (Bužančić et al., 2022).

There are many decision-support tools for guiding deprescribing and promoting medication safety, such as databases for drug-drug interactions (DDIs) and ADRs as well as PIM lists. The most commonly used and relevant PIM lists in the Eastern European region are the European List of Potential Inappropriate Medications [EU(7)-PIM] and EURO-FORTA (Renom-Guiteras et al., 2015; Pazan et al., 2018). The International Pharmaceutical Federation MR toolkit encourages the use of PIM lists and other decision-support systems in tandem with patient interviews as they are useful tools in guiding pharmacists when conducting MRs (International Pharmaceutical Federation FIP, 2022).

Pharmacist-led MRs aimed at polypharmacy patients have been implemented in most Northern and Western European countries for many years, but this has not been the case in Eastern Europe until recently (Bulajeva et al., 2014; Soares et al., 2020). Although pharmacist integration onto the healthcare team has been slow in the region, there is a clear need for services aimed at medication optimization and safety. The prevalence of multimorbidity is much higher in Eastern Europe when compared to other regions in Europe and is the highest among elderly patients in Hungary and Estonia (Palladino et al., 2016; Nielsen et al., 2017; Midão et al., 2018; Kardas et al., 2021). The prevalence of polypharmacy in Europe is between 26.3% and 39.9% and has been found to be 33.8% in Poland and 28.4% in Estonia (Midão et al., 2018). However, these rates are higher in patients aged ≥85—up to 57.0% for Poland, 41% for Bulgaria, and 31.6% for Estonia (Midão et al., 2018; Krustev et al., 2022a). Additionally, potentially inappropriate prescribing (PIP), which includes prescribing potentially inappropriate medicines and potential prescribing omissions, has been noted as a serious problem among elderly patients in Bulgaria, where in every 78 prescriptions, there is a chance of PIP (Krustev et al., 2022b). The estimated overall PIP prevalence is 22.6%; however, it could be higher in Eastern and Central Europe—around 34.6% (Tommelein et al., 2015; Brkic et al., 2022). Due to an aging population, polypharmacy and inappropriate prescribing are expected to rise in the Eastern European region. While there are barriers at the healthcare policy and organizational levels, community pharmacists are well placed and willing to offer MRs to promote the rational use of medicines and benefit patients (Tuula et al., 2021; Merks et al., 2022; Paidere et al., 2023).

The aim of this study was to pilot MRs in Eastern European community pharmacies and evaluate the usability of medication safety assessment tools in order to promote the development and implementation of a patient safety-oriented community pharmacy-based MR service.

## 2 Methods

### 2.1 The MR pilot project in Eastern Europe

In September 2017, the Eastern European MR pilot project standards were established in Estonia. The service was intended as an intermediate type 2A/2B MR, based on the Pharmaceutical Care Network Europe statement (2013), conducted in a community pharmacy by a pharmacist, where the pharmacist receives information about the treatment regimen and patient’s diseases from both the patient in a face-to-face interview and their general practitioner (GP). However, in most cases, the pharmacist does not have access to clinical data such as results of blood tests. Participating pharmacists were not required to go through a clinical pharmacy or medication review certification program prior to the pilot but were introduced to the aims and structure of the service by project coordinators. Participating pharmacists did not get remunerated for offering the service.

**FIGURE 1:**
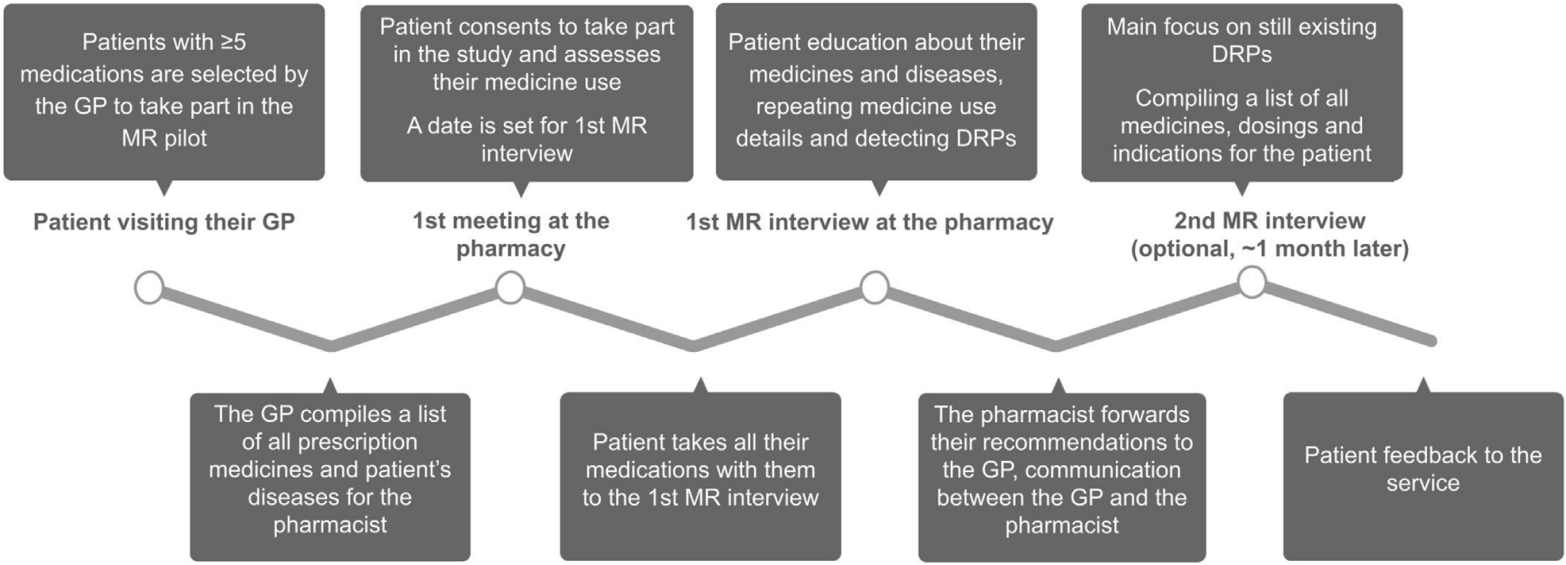
Eastern European MR pilot project structure.

Patients were recruited to the project by their general practitioner; the only inclusion criterion was that the patient had to be using five or more medicines. The GP compiled a list of all the patient’s diseases and prescription medications, which was shared with the pharmacist. The patient then turned to the project pharmacy, gave their consent to take part in the study, registered for the first interview, and evaluated their medication use. For the first interview, the patient was instructed to bring all their medicines, food supplements, and herbal products with them. Structured patient interviews were conducted to collect data on the patient’s medication use and adherence to their treatment plan. The aim of the service was to educate the patient on their diseases and medicines and to detect medication non-adherence and DRPs. All participants took part in the first MR interview and were invited to the follow-up interview, if necessary. After the last interview, the pharmacist forwarded their recommendations to the GP and compiled a table for the patient, including the names of all their medicines, instructions for use, and indications. The patient gave feedback on the service. The pilot service structure is depicted in Figure 1. Structured interview forms can be found under Supplementary Material.

In January 2019, the pilot started in Estonia. Soon after, an Eastern European working group was formed with researchers from eleven countries: Estonia, Latvia, Lithuania, Poland, Slovakia, Hungary, Croatia, Bosnia and Herzegovina, Romania, Bulgaria, and Iran. The pilot was completed according to the agreed protocol by the summer of 2021 in six of the eleven countries, namely,: Estonia, Latvia, Poland, Hungary, Romania, and Bulgaria.

Only data from the first interview have been included in this study, as not all patients needed a second interview.

### 2.2 Inxbase/Riskbase

To determine pDDIs and potential ADRs in the study, population databases Inxbase/Riskbase were used retroactively. These databases were selected because they are available for community pharmacists in Estonia (Tuula et al., 2022). Inxbase divides pDDIs into four categories based on the risk to health: D—high risk, combination should be avoided; C—moderate risk, combination can be used but dose adjustment might be needed; B—unclear risk; A—clinically insignificant risk (Inxbase/Riskbase, 2019).

Riskbase divides the potential ADRs into ten groups: bleeding risk, constipation, anticholinergic effect, orthostatism, prolonging of the QT interval, renal toxicity, sedation, seizure risk, serotonergic effect, and potassium level. By taking into consideration the entire treatment plan, Riskbase calculates a risk score in all 10 groups as follows: D—high risk; C—moderate risk; B—slightly increased risk; A—no known risk. The high-risk category on Riskbase does not contraindicate using the medicine but defines the probability of risk (Inxbase/Riskbase, 2019).

Analysis based on Inxbase/Riskbase was applied retroactively to treatment regimens for all participating patients and compared with pharmacist notes on DRPs related to DDIs and ADRs documented during the service. The authors took into consideration the doses of the medicine, dosage form and dosing frequency when determining the risk with Inxbase/Riskbase (e.g., non-steroid anti-inflammatory drugs were not included in the analysis if they were in topical drug formulations or used *ad hoc* less than once a week or, in the case of low-dose aspirin, when the database indicated no similar risks in smaller doses).

### 2.3 EU(7)-PIM/EURO-FORTA combined PIM list

For this study, the integrated tool of databases EU (7)-PIM (Renom-Guiteras et al., 2015) and EURO-FORTA (Pazan et al., 2018) was used to evaluate medication safety in elderly participants retroactively (Bobrova et al., 2022). With this tool, PIMs are classified as follows:

- high-risk PIMs, which should be avoided in elderly patients
- moderate-risk PIMs, which may require dose or treatment duration adjustment
- low-risk PIMs, which rarely cause DRPs that are concerning in older patients specifically (Bobrova et al., 2022)

The tool also uses color coding for PIMs as follows:

- red = high-risk PIM, which is included in both EURO-FORTA and EU(7)-PIM
- yellow = medium-risk PIM, which is included in both EURO-FORTA and EU(7)-PIM
- green = low-risk PIM, which is included in both EURO-FORTA and EU(7)-PIM
- gray = high, medium or low-risk PIM depending on circumstances, but the medicine is not included in either EURO-FORTA or EU(7)-PIM; therefore, there is less evidence of its inappropriateness (Bobrova et al., 2022)

The list takes into consideration treatment indication, drug dosage, and other factors determining a PIM from the original lists (Bobrova et al., 2022).

Only patients aged 65 or older were included in this part of the study. To conduct the analysis, all active substances in the treatment regimen of elderly participants were coded based on the combined tool. Medicines were only considered PIMs if they met the given EU(7)-PIM and EURO-FORTA criteria (e.g., dosage and indication). High and medium-risk PIM data were compared retroactively to DRP data collected during the MR pilot.

## 3 Results

### 3.1 Overview of study participants

A total of 318 polypharmacy patients were included in the study from six countries. Two hundred and fifty (79%) of the participants were 65 years or older. On average, the first interview with the patient took 33.6 min. The mean age of the participants was 71.2 ± 10.9, with 59.9% of participants being female and 40.1% male. The participants had on average 4.3 ± 2.0 conditions, most commonly diseases of the circulatory system and endocrine, nutritional, and metabolic diseases based on the ICD-10 classification. The median number of medicines per patient was 7, with the maximum being 22 for one patient in Poland. The most commonly used medicines included ACE inhibitors, beta-blockers, and statins. A description of the study population by country can be found in Table 1.

**TABLE 1:** Description of the study population by country.

Country	Number of participants (% of total participants) N = 318	Number of elderly participants (% of total elderly participants) N = 250	Median number of medicines used by a participant (max)	Median number of DRPs detected per patient (range)
Estonia	66 (20.8%)	48 (19.2%)	8 (19)	2 (0–8)
Latvia	23 (7.2%)	17 (6.8%)	8 (16)	2 (0–7)
Poland	48 (15.1%)	32 (12.8%)	6.5 (22)	2 (0–8)
Hungary	55 (17.3%)	52 (20.8%)	10 (17)	1 (0–9)
Romania	56 (17.6%)	48 (19.2%)	6 (10)	2 (0–7)
Bulgaria	70 (22.0%)	53 (21.2%)	5 (8)	1 (0–6)

### 3.2 Inxbase-based potential drug-drug interaction analysis

In total, 318 treatment regimens were analyzed using the Inxbase pDDI database. Of the 318 participants, 180 (56.6%) had at least one pDDI. Altogether, 504 interactions were detected—on average 1.6 per study participant. The maximum number of pDDIs according to Inxbase was 12 for one participant from Estonia.

**FIGURE 2:**
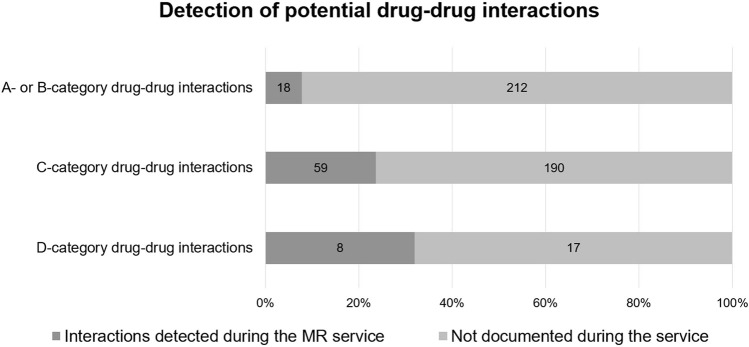
Potential drug-drug interactions according to Inxbase (*n* = 504) compared to drug-drug interactions documented during the MR service (*n* = 85).

While more than 70% of participants from Estonia and Hungary had at least one pDDI according to Inxbase, this percentage was much lower in Bulgaria (28.6%), most likely due to the participants having fewer medicines on average in their treatment regimens. Nineteen (6.0%) of the total 318 patients had at least one D-category pDDI according to Inxbase. The number of participants with D-category pDDIs was the highest in Hungary (8/55), Estonia (5/66), and Poland (3/48). Twenty-five (5.0%) pDDIs of the total 504 were D-category interactions, indicating that the given combination of medicines should be avoided. Higher category pDDIs were more likely to be detected and documented during the service (see Figure 2). Of the 504 pDDIs provided by Inxbase, 85 (16.9%) were detected and documented during the service.

Twenty-seven DDIs, which were not listed on the Inxbase database, were documented during the service by the pharmacists. Some of them involved medicines and herbal supplements not listed on the Inxbase database at the time (e.g., nimesulide and grapefruit preparations). The highest number of DDIs not on the database was documented in Poland (15 all together).

The most common pDDIs were: the decrease in effectiveness and decline in kidney function caused by the use of antihypertensives with NSAIDs on a daily basis; and the risk of hyperkalemia when combining spironolactone with ACE inhibitors, ARBs, or potassium supplements. The most common high risk pDDI was the increased risk of bleeding due to the use of an antithrombotic medicine (e.g., apixaban, rivaroxaban, warfarin, dabigatran) with medicines increasing their effect or causing bleeding as a side effect. The latter were also detected most often by pharmacists as DRPs during the service.

### 3.3 Riskbase-based cumulative adverse effect analysis

For the 318 treatment regimens analyzed, 735 potential ADRs were detected in ten categories (bleeding risk, constipation, anticholinergic effect, orthostatism, prolonging of the QT interval, renal toxicity, sedation, seizure risk, serotonergic effect, and potassium balance). Two hundred and seventy-nine (87.7%) of the participants had at least a B category side effect in at least one of the ten categories. On average, 2.3 potential ADRs were detected per study participant.

**FIGURE 3:**
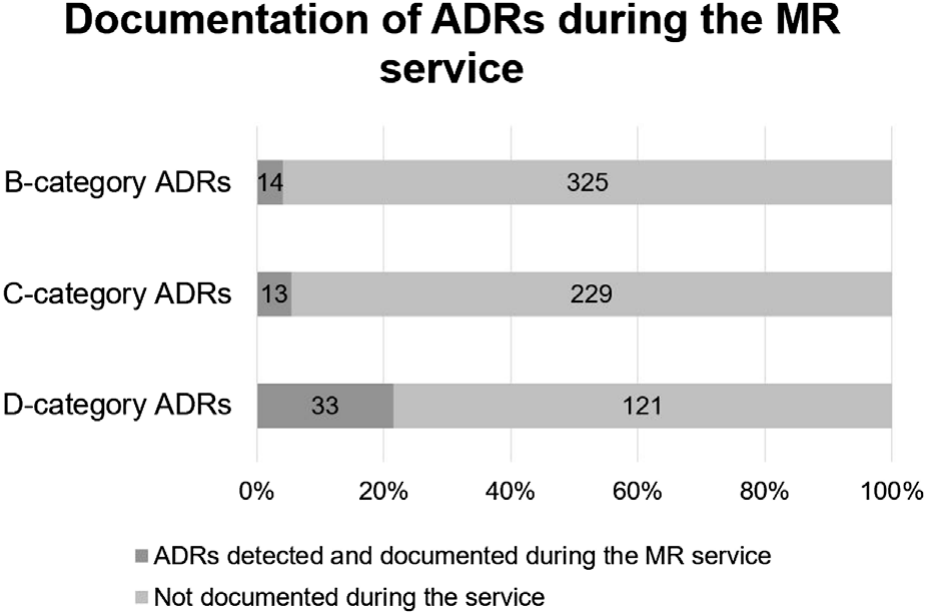
Comparison of Riskbase provided potential ADRs (*n* = 735) and ADRs detected by pharmacists (*n* = 60).

Compared to pDDIs, many more potential D-category risks were identified with Riskbase—154 in 110 participants. Again, higher category risks were more likely to be documented at the MR service, but less often than for pDDIs (see Figure 3). This might mean that the feasibility of use of Riskbase is lower for MRs or that pharmacists are less likely to monitor for potential ADRs than for pDDIs.

The number of participants with potential D-category ADRs was again the highest in Estonia (38/66), Hungary (27/55), and Poland (21/48). One patient from Estonia had at least a B-category risk in all ten Riskbase subcategories.

The most common potential ADR for the participants was constipation, with 208 (65.4%) study participants having at least a B-category risk for constipation. Sixty-eight other side effects not stated in any of the 10 Riskbase categories were documented by pharmacists during the MR service, which were most commonly cough caused by an ACE inhibitor and oral fungal infections caused by inhaled corticosteroids.

### 3.4 EU(7)-PIM/EURO-FORTA integrated PIM list-based analysis

A total of 2037 medicines were used by the 250 elderly participants in the study. Eight hundred and sixteen (40%) of all medicines used by the older patients were potentially inappropriate according to the EU(7)-PIM/EURO-FORTA combined PIM list. Differences in PIM use per project country are depicted in Table 2.

**TABLE 2:** Potentially inappropriate medicines use in elderly participants (*n* = 250) in the different project countries.

Country	Number of elderly participants	Total number of PIMs detected (range per participant)	Number of elderly participants using any category PIM (%)	Number of elderly participants using either high or medium risk PIMs (%)	Number of elderly participants using at least one high risk PIM (%)
Estonia	48	206 (0–9)	47 (97.9%)	37 (77.1%)	18 (37.5%)
Latvia	17	50 (1–6)	17 (100%)	8 (47.1%)	6 (35.3%)
Poland	32	89 (0–9)	27 (84.4%)	18 (56.3%)	9 (28.1%)
Hungary	52	203 (1–8)	52 (100%)	34 (65.4%)	17 (32.7%)
Romania	48	132 (0–5)	46 (95.8%)	27 (56.3%)	15 (31.3)
Bulgaria	53	136 (0–5)	51 (96.2%)	22 (41.5%)	6 (11.3%)
**Total**	**250**	**816 (0–9)**	**240 (96.0%)**	**146 (58.4%)**	**71 (28.4%)**

The bold values represent the number of elderly patient etc for the whole study population (participants from all countries).

High-risk PIMs made up 85 of the 816 PIMs (10.4%), with 73 (85.9%) being described in both PIM databases (high-risk red PIMs) and 12 (14.1%) being present in one or the other (high-risk gray PIMs). NSAIDs constituted 37.6% of high-risk PIMs, benzodiazepines 23.5%, and statins 10.6%, making them the most used high-risk PIMs in the study population (N = 250). The rate of high-risk PIMs per elderly participant of each country was the highest in Estonia (0.50) and Hungary (0.44), and lowest in Bulgaria (0.15) and Poland (0.28). Eighteen (21.2%) of the high-risk PIMs were also recorded as DRPs in the MR service documentation.

Medium-risk PIMs constituted 152 of the 816 PIMs (19.2%). In this regard, 101 (66.5%) were described in both PIM databases (medium-risk yellow PIMs) and 51 (33.6%) were present in one or the other (medium-risk gray PIMs). The most commonly used medium-risk PIMs in the study population (N = 250) were Z-drugs (12.5%), nitrates (11.8%), tramadol (9.9%), moxonidine (9.9%), spironolactone (7.2%), and pregabalin/gabapentin (6.6%). The rate of medium-risk PIMs per elderly participant of each country was again the highest in Estonia (0.86) and Hungary (0.79), and lowest in Bulgaria (0.42) and Romania (0.40). Twenty-one (13.8%) of the medium-risk PIMs were also recorded as DRPs in the MR documentation.

Low-risk PIMs made up 579 of the 816 PIMs (70.9%), with 317 (54.8%) being described in both databases (low-risk green PIMs) and 262 (45.2%) being present in one or the other (low-risk gray PIMs).

## 4 Discussion

Standardization of MR practices is considered important, both to ensure high quality service provision and to promote research on MR outcomes. Assessment of possible ADRs, pDDIs, and appropriateness is considered essential to an MR service, yet tools for conducting the assessment vary in their reliability and usability. Although Rose O et al. refer to standardization of the service on an international scale, national or region-based standardization is needed for similar reasons, including tools for the service (Rose et al., 2020). For this study, the recruited pharmacists were free to use the tools they use in their everyday practice. However, the service could greatly benefit from regularly updated and reliable clinical decision-support tools, which are available and remunerated for all pharmacists conducting MRs. Training on how to use different decision-support systems is recommended.

Previous studies using databases for pDDIs on MR results show variation between the number and the risk level of pDDIs identified. For example, one study conducted in Germany on home-dwelling patients found 3,025 pDDIs in 779 participants (3.9 per participant). However, only eleven (0.4%) were seriously clinically relevant and 1,326 (43.8%) moderately relevant (Hoffmann et al., 2011). As the participants of the study are similar in age and number of medicines to the study population in our study, the results can be compared. The proportions of moderate and high risk pDDIs were slightly higher based on the Inxbase analysis (5.0% for high risk pDDIs and 49.4% of medium risk pDDIs), yet less pDDIs were detected (1.6 per participant). Large differences in content for local medication safety databases can hinder research on medication safety, as they set dissimilar standards for risks.

It is important to not rely on decision-support tools alone when conducting MRs. Firstly, MRs involve medication reconciliation as the first step of the service. Without gathering information about the full medication list, including over-the-counter medicines, food supplements and herbal products, the analysis of medication safety is incomplete. Moreover, tools for evaluating medication safety cannot replace patient interviews, as the potential risks of these tools have been known to far outnumber the risks that manifest as ADEs (Magro et al., 2012). Not all risks are relevant for all patients, e.g., a risk of increase in potassium levels might be harmful for one patient but an expected outcome of treatment for another. Our study results align with these previous findings, as only a fraction of the potential risks provided by Inxbase/Riskbase were detected by pharmacists during MRs (16.9% for pDDIs and 8.2% for ADRs). To evaluate the manifestation of potential risks, patient interviews proved very effective. ADRs (such as statin-induced muscle ache or ACE inhibitor-related cough) are especially difficult to predict even when using a decision-support system and impossible to monitor without discussing health complaints with the patient. However, when using databases such as Inxbase/Riskbase, a patient’s clinical information is often necessary to evaluate the likelihood of the risk manifesting. Thus, to support medication safety, the authors consider patient interviews, preferably conducted by pharmacists, in addition to access to all clinical data, essential in delivering high quality MRs.

According to our study, 32% of D-category pDDIs and 21.4% of D-category potential ADRs were documented during the service as DRPs. These high risks indicate a need for deprescribing or modifying the treatment plan. Based on this, recommendations were forwarded to GPs by the pharmacists. To evaluate the effectiveness of the service, it would be beneficial to collect information on the acceptance of the pharmacist’s solution and the final status of the DRP, e.g., using the Pharmaceutical Care Network Europe DRP classification system as a base model for the analysis of results (Pharmaceutical Care Network Europe, 2019). However, in this study, the authors lacked information on the results of consultations with the patient’s GP. Moreover, during the pilot, most pharmacists consulted GPs over the phone, which is not an efficient way of sharing information between healthcare workers, as some important details of the MR results can go missing. Follow-up consultations with the patient to collect information on the final status of the DRP are essential for understanding the clinical value of the service. Simple phone-based patient consultations could increase the participation rate for follow-ups, as many patients will not require a thorough second interview at the pharmacy.

Using PIM lists in pharmacist-led MRs comes with a number of obstacles. It has previously been noted that PIM lists are inconvenient to use, hard to find, and difficult to keep up to date due to their being available only through Supplementary Material of original research papers in PDF format (Bobrova et al., 2022). In addition, EU(7)-PIM and EURO-FORTA come with very little detail and sometimes with no description at all on the risks associated with the use of certain PIMs in elderly patients, which greatly restricts their use in both practice and research. When comparing PIM data to DRPs documented during the study, for some items, the PIM lists did not provide sufficient information for proper comparison of risks. For example, fluticasone is a medium-risk PIM, especially in elderly COPD patients, according to EURO-FORTA. However, neither of the PIM lists specifies the reason for the inclusion of fluticasone, which makes it difficult to screen for the manifestation of the potential risk or measure it in research. An interactive electronic format for PIMs would improve the usability and accessibility and would not restrict the length of the content as much. Thus, there is a need to update the decision-support systems commonly used in pharmacy practice with PIM lists in order to support their use in MRs.

Another barrier to the use of PIM lists in pharmacy practice is the high number of low-risk PIMs, which rarely cause DRPs specific to elderly patients, thus the lists are greatly overpopulated. A previous study from Lithuania showed substantial differences in PIM rates when the data for elderly patients were analyzed with 2015 AGS Beers criteria compared to EU (7)-PIM—25.9% vs. 57.2%, respectively (Grina and Briedis, 2017). In our study, 96% of older polypharmacy participants were using at least one PIM, most of them low-risk PIMs such as thiazide diuretics, metformin, and proton pump inhibitors. PIMs have been defined as medicines that cause more harm than good in older patients (Malakouti et al., 2021). Due to the lack of safer alternatives and the absence of recommendations for improving safety, the value of including these medicines in PIM lists is questionable and hinders the use of such lists in practice. Although EU(7)-PIM is considered the most appropriate PIM list to use in the European region, a shortened version only including high and medium-risk PIMs could be beneficial for MRs.

Another aim of this study was to give an overview of medication use in mostly elderly polypharmacy patients in Eastern European countries. Although some isolated studies have been conducted on the topic, very few focus on the region as a whole. The authors encourage regional collaboration in research focused on medication use and improving medication safety through pharmacy services.

## 5 Conclusion

The need for MRs in Eastern Europe is evident, as polypharmacy is on the rise. Based on the study, elderly polypharmacy patients in the region often use PIMs. More than half of the participants had pDDI, with a high proportion of them being medium or high risk. Further intervention to improve medication safety in this patient group is thus important.

The first successful testing of a pharmacist-led MR service has been completed in Estonia, Latvia, Poland, Hungary, Bulgaria, and Romania, but further steps need to be taken to fully implement the service in the region. Decision-support tools for detecting possible ADEs can guide decision-making and aid in patient monitoring; however, they cannot replace patient interviews and medication reconciliation. Our study indicates that the pDDI database Inxbase and ADR database Riskbase can be useful in guiding the interviews and monitoring risks. The pharmacists participating in the study only documented a few problems regarding pDDIs and ADRs that the databases did not include. It should be a national healthcare priority not only to create and update such databases, but also to promote the use of them as standard tools. Accessibility and remuneration of medication safety assessment tools for pharmacists, as well as previous training on the use of such tools are preconditions to support the consistent quality of the MR service.

PIM lists could also be useful for MR services, especially considering the prevalence of high and medium-risk PIM use in our study population. Although available for all, the PIM lists used in this study are currently impractical for MRs due to their inconvenient form and lack of explanation on the associated risks.

### 5.1 Limitations

The main limitation of this study is access to the databases Inxbase and Riskbase. These databases are currently only available for limited groups of health workers in Estonia and are not intended for international use. Another limitation is that no obligatory course was outlined for the service providers on the clinical aspect of the project, nor were all service providers using the same tools to analyze the drug-related problems. The results can be biased due to interindividual differences when determining DDIs and ADRs.

## Data Availability

The datasets presented in this article are not readily available because they include information from the Inxbase/Riskbase database, which are not publicly available. Requests to access the datasets should be directed to anita.tuula@ut.ee.
