# Supplementary material for: Evaluation of medication safety assessment tools for pharmacist-led medication reviews: the Eastern European pilot project
Source: Front Pharmacol. 2024 Feb 16;15:1348400. doi: 10.3389/fphar.2024.1348400 (PMC10904472; doi:10.3389/fphar.2024.1348400)
Supplement: Supplementary file 1 [file DataSheet1.PDF]

Patient code: Date of registration:

1. Patient information

First name

Last name

Age

Gender
- ☐ Male
- ☐ Female

General practitioner

Contact information

2. Health information

Weight

Height

BMI

Allergies

Diet

Physical activity

Smoking
- ☐ Yes
- ☐ No

If yes, how many cigarettes a day?

Alcohol consumption
- ☐ Yes
- ☐ No

If yes, how often?
- ☐ About once a month or less
- ☐ Two to four times a month
- ☐ Two to four times a week
- ☐ Four or more times a week

How many units of alcohol is usually consumed?

Patient code: Date of registration:

3. Patient self-evaluation of medicines use and perception of medicines

Rate the following statements on a 5-point scale.

My health situation is…

☐ Very poor ☐ Poor ☐ Fair ☐ Good ☐ Very good

I understand my disease(s).

☐ Strongly disagree ☐ Disagree ☐ Neither agree nor disagree ☐ Agree ☐ Strongly agree

I am often concerned about the use of my medicines.

☐ Strongly disagree ☐ Disagree ☐ Neither agree nor disagree ☐ Agree ☐ Strongly agree

My health situation depends on my medicines.

☐ Strongly disagree ☐ Disagree ☐ Neither agree nor disagree ☐ Agree ☐ Strongly agree

Without medicines I would be very sick.

☐ Strongly disagree ☐ Disagree ☐ Neither agree nor disagree ☐ Agree ☐ Strongly agree

I do not understand how my medicines improve my health.

☐ Strongly disagree ☐ Disagree ☐ Neither agree nor disagree ☐ Agree ☐ Strongly agree

Taking tablets is easy.

☐ Never ☐ Rarely ☐ Sometimes ☐ Often ☐ Very often

I forget to take my medicines.

☐ Never ☐ Rarely ☐ Sometimes ☐ Often ☐ Very often

I have difficulties getting my medicines out of the packaging.

☐ Never ☐ Rarely ☐ Sometimes ☐ Often ☐ Very often

I mix up different medicines.

☐ Never ☐ Rarely ☐ Sometimes ☐ Often ☐ Very often

I have problems with finding my medicines.

☐ Never ☐ Rarely ☐ Sometimes ☐ Often ☐ Very often

Patient code: Date of registration:

I have difficulties with swallowing tablets.

☐ Never ☐ Rarely ☐ Sometimes ☐ Often ☐ Very often

I have difficulties with remembering the doses of my medicines.

☐ Never ☐ Rarely ☐ Sometimes ☐ Often ☐ Very often

I do not have enough money to pay for my medicines.

☐ Never ☐ Rarely ☐ Sometimes ☐ Often ☐ Very often

I have experienced adverse effects when taking my medicines.

☐ Never ☐ Rarely ☐ Sometimes ☐ Often ☐ Very often

I am concerned about the consequences of long-term use of medicines.

☐ Never ☐ Rarely ☐ Sometimes ☐ Often ☐ Very often

Taking medicines has caused some other concerns or problems for me.

☐ Never ☐ Rarely ☐ Sometimes ☐ Often ☐ Very often

4. Documentation of the 1st MR interview

Date of the interview:

Patient's diseases (information both from GP and patient)

1) Disease
ICD-10 code

2) Disease
ICD-10 code

3) Disease
ICD-10 code

4) Disease
ICD-10 code

5) Disease
ICD-10 code

6) Disease
ICD-10 code

7) Disease
ICD-10 code

8) Disease
ICD-10 code

Patient's medicines and food supplements (information both from GP and patient)

1) Medicine trade name
Active ingredient
Dosage form (e.g. tablets, capsules)
Route of administration and administration details
Additional notes

2) Medicine trade name
Active ingredient
Dosage form (e.g. tablets, capsules)
Route of administration and administration details
Additional notes

3) Medicine trade name
Active ingredient
Dosage form (e.g. tablets, capsules)
Route of administration and administration details
Additional notes

4) Medicine trade name
Active ingredient
Dosage form (e.g. tablets, capsules)
Route of administration and administration details
Additional notes

5) Medicine trade name
Active ingredient
Dosage form (e.g. tablets, capsules)
Route of administration and administration details
Additional notes

6) Medicine trade name
Active ingredient
Dosage form (e.g. tablets, capsules)
Route of administration and administration details
Additional notes

7) Medicine trade name
Active ingredient
Dosage form (e.g. tablets, capsules)
Route of administration and administration details
Additional notes

8) Medicine trade name
Active ingredient
Dosage form (e.g. tablets, capsules)
Route of administration and administration details
Additional notes

9) Medicine trade name
Active ingredient
Dosage form (e.g. tablets, capsules)
Route of administration and administration details
Additional notes

Drug-related problems

DRP classification:

- ☐ Patient does not know the indication of the medicine
- ☐ Patient alters their medication regimen
- ☐ Patient omits doses
- ☐ Medicine is administered at the wrong time
- ☐ Difficulties with swallowing
- ☐ Adverse effect
- ☐ Drug interaction
- ☐ Other

Describe the problem and solution in detail

DRP classification:

- ☐ Patient does not know the indication of the medicine
- ☐ Patient alters their medication regimen
- ☐ Patient omits doses
- ☐ Medicine is administered at the wrong time
- ☐ Difficulties with swallowing
- ☐ Adverse effect
- ☐ Drug interaction
- ☐ Other

Describe the problem and solution in detail

Patient code: Date of registration:

DRP classification:	Describe the problem and solution in detail
☐ Patient does not know the indication of the medicine<br>☐ Patient alters their medication regimen<br>☐ Patient omits doses<br>☐ Medicine is administered at the wrong time<br>☐ Difficulties with swallowing<br>☐ Adverse effect<br>☐ Drug interaction<br>☐ Other	

DRP classification:	Describe the problem and solution in detail
☐ Patient does not know the indication of the medicine<br>☐ Patient alters their medication regimen<br>☐ Patient omits doses<br>☐ Medicine is administered at the wrong time<br>☐ Difficulties with swallowing<br>☐ Adverse effect<br>☐ Drug interaction<br>☐ Other	

Notes and recommendations from the pharmacist to the GP

Notes and recommendations from the pharmacist to the patient/caregiver

Time of the next interview (if needed):

Reason for the next interview

Time of the interview in minutes:

Time of preparation in minutes:

5. Documentation of the 2nd MR interview

Date of the interview:

Patient’s diseases (information both from GP and patient)

1) Disease
ICD-10 code

2) Disease
ICD-10 code

3) Disease
ICD-10 code

4) Disease
ICD-10 code

5) Disease
ICD-10 code

6) Disease
ICD-10 code

7) Disease
ICD-10 code

8) Disease
ICD-10 code

Patient’s medicines and food supplements (information both from GP and patient)

1) Medicine trade name
Active ingredient
Dosage form (e.g. tablets, capsules)
Route of administration and administration details
Additional notes

2) Medicine trade name
Active ingredient
Dosage form (e.g. tablets, capsules)
Route of administration and administration details
Additional notes

3) Medicine trade name
Active ingredient
Dosage form (e.g. tablets, capsules)
Route of administration and administration details
Additional notes

4) Medicine trade name
Active ingredient
Dosage form (e.g. tablets, capsules)
Route of administration and administration details
Additional notes

Patient code: Date of registration:

5) Medicine trade name
Active ingredient
Dosage form (e.g. tablets, capsules)
Route of administration and administration details
Additional notes

6) Medicine trade name
Active ingredient
Dosage form (e.g. tablets, capsules)
Route of administration and administration details
Additional notes

7) Medicine trade name
Active ingredient
Dosage form (e.g. tablets, capsules)
Route of administration and administration details
Additional notes

8) Medicine trade name
Active ingredient
Dosage form (e.g. tablets, capsules)
Route of administration and administration details
Additional notes

9) Medicine trade name
Active ingredient
Dosage form (e.g. tablets, capsules)
Route of administration and administration details
Additional notes

Drug-related problems

DRP classification:

- ☐ Patient does not know the indication of the medicine
- ☐ Patient alters their medication regimen
- ☐ Patient omits doses
- ☐ Medicine is administered at the wrong time
- ☐ Difficulties with swallowing
- ☐ Adverse effect
- ☐ Drug interaction
- ☐ Other

Describe the problem and solution in detail

DRP classification:

- ☐ Patient does not know the indication of the medicine
- ☐ Patient alters their medication regimen
- ☐ Patient omits doses
- ☐ Medicine is administered at the wrong time
- ☐ Difficulties with swallowing
- ☐ Adverse effect
- ☐ Drug interaction

Describe the problem and solution in detail

- ☐ Other

DRP classification:	Describe the problem and solution in detail
☐ Patient does not know the indication of the medicine<br>☐ Patient alters their medication regimen<br>☐ Patient omits doses<br>☐ Medicine is administered at the wrong time<br>☐ Difficulties with swallowing<br>☐ Adverse effect<br>☐ Drug interaction<br>☐ Other	

DRP classification:	Describe the problem and solution in detail
☐ Patient does not know the indication of the medicine<br>☐ Patient alters their medication regimen<br>☐ Patient omits doses<br>☐ Medicine is administered at the wrong time<br>☐ Difficulties with swallowing<br>☐ Adverse effect<br>☐ Drug interaction<br>☐ Other	

Notes and recommendations from the pharmacist to the GP

Notes and recommendations from the pharmacist to the patient/caregiver

Time of the interview in minutes:

Time of preparation in minutes:

Patient code: Date of registration:

6.	**Patient's feedback to the MR service after the last interview**			
I am satisfied with the pharmacist's explanation of the aims of the MR service				
☐ Strongly disagree	☐ Disagree	☐ Neither agree nor disagree	☐ Agree	☐ Strongly agree
I am satisfied with the privacy and comfort in the consultation room.				
☐ Strongly disagree	☐ Disagree	☐ Neither agree nor disagree	☐ Agree	☐ Strongly agree
I am satisfied with the pharmacist's personal approach.				
☐ Strongly disagree	☐ Disagree	☐ Neither agree nor disagree	☐ Agree	☐ Strongly agree
I am satisfied with the opportunity to raise questions or concerns.				
☐ Strongly disagree	☐ Disagree	☐ Neither agree nor disagree	☐ Agree	☐ Strongly agree
I am satisfied with the pharmacist's advice and recommendations				
☐ Strongly disagree	☐ Disagree	☐ Neither agree nor disagree	☐ Agree	☐ Strongly agree
The pharmacist answered my questions.				
☐ Strongly disagree	☐ Disagree	☐ Neither agree nor disagree	☐ Agree	☐ Strongly agree
I feel more confident in managing my condition.				
☐ Strongly disagree	☐ Disagree	☐ Neither agree nor disagree	☐ Agree	☐ Strongly agree
I know when and how to take my medications				
☐ Strongly disagree	☐ Disagree	☐ Neither agree nor disagree	☐ Agree	☐ Strongly agree
I know how important it is to take medications regularly.				
☐ Strongly disagree	☐ Disagree	☐ Neither agree nor disagree	☐ Agree	☐ Strongly agree
I would take part in the MR service in the future.				
☐ Strongly disagree	☐ Disagree	☐ Neither agree nor disagree	☐ Agree	☐ Strongly agree

Patient code: Date of registration:

I would recommend the MR service to others.

- ☐ Strongly disagree
- ☐ Disagree
- ☐ Neither agree nor disagree
- ☐ Agree
- ☐ Strongly agree

I am satisfied with the MR service I took part in.

- ☐ Strongly disagree
- ☐ Disagree
- ☐ Neither agree nor disagree
- ☐ Agree
- ☐ Strongly agree
